# Divergent evolutionary patterns of the MAPK cascade genes in *Brassica rapa* and plant phylogenetics

**DOI:** 10.1038/hortres.2017.79

**Published:** 2017-12-27

**Authors:** Peng Wu, Wenli Wang, Ying Li, Xilin Hou

**Affiliations:** 1 State Key Laboratory of Crop Genetics and Germplasm Enhancement/Key Laboratory of Biology and Germplasm Enhancement of Horticultural Crops in East China, Ministry of Agriculture, College of Horticulture, Nanjing Agricultural University, Nanjing 210095, China

## Abstract

Mitogen-activated protein kinase (MAPK) cascade signal transduction modules play crucial roles in regulating many biological processes in plants. These cascades are composed of three classes of hierarchically organized protein kinases, MAPKKKs, MAPKKs and MAPKs. Here, we analyzed gene retention, phylogenetic, evolution and expression patterns of MAPK cascade genes in *Brassica rapa*. We further found that the MAPK branches, classes III and IV, appeared after the split of bryophytes and green algae after analyzing the MAPK cascade genes in 8 species, and their rapid expansion led to the great size of the families of MAPKs. In contrast, the ancestral class I subfamily of MAPKK gene families have been highly conserved from algae to angiosperms. For the MAPKKK family, the MEKK and Raf subfamily share a common evolutionary origin, and Raf plays a major role in the expansion of the MAPKKK gene family. The *cis*-elements and interaction network analyses showed the important function of MAPK cascade genes in development and stress responses in *B. rapa*. This study provides a solid foundation for molecular evolution analyses of MAPK cascade genes.

## Introduction

Based on their exposure to environmental stress, plants have constructed complex signaling networks to adapt to different stress conditions.^1^ Protein kinases (PKs) play important roles in both growth and reproduction processes.^1,2^ As a highly conserved sub-family of PKs, the mitogen-activated protein kinases (MAPKs) consist of the following three classes: (1) MAPKs, (2) MAPK kinases (MAPKKs), and (3) MAPKK kinases (MAPKKKs), which act as a signal transmission cascade in the cellular metabolic and transcriptional response centers.^1,2^ MAPKs are activated by MAPKKs via phosphorylation of conserved threonine and tyrosine residues in the Thr–X–Tyr (T–X–Y) motif. MAPKKs, in turn, are activated by MAPKKKs when serine and threonine residues in the S/TXXXXXS/T motif are phosphorylated.^3–9^

In eukaryotic organisms, MAPK pathways are relatively well-characterized cascades.^10^ In plants, genome-wide analyses of MAPK cascades (MAPKKK—MAPKK-MAPK) have been reported in several species.^11–18^ In *Arabidopsis*, 23 MAPKs, 10 MAPKKs, and 80 putative MAPKKKs have been identified. MAPK and MAPKK genes in plants are each divided into four distinct classes (classes A, B, C, and D), whereas MAPKKK genes are classified into the following three subfamilies: (1) MEKK, (2) Raf and (3) ZIK.

Plant MAPKs are involved in both biotic stresses, such as AtMAPK4,^18,19^ MsMMK2, and MsMMK3,^1^ and in abiotic stresses, such as AtMAPK4,^20^ OsMAPK4 ^21^ and AtMAPK7.^22^ MKK9-MPK3/MPK6 regulates ethylene signaling and may also play a role in leaf senescence.^9,23,24^ Plant MKKs(MAPKKs) contain a phosphorylation site (S/T-X5-S/T) and a putative MAPK docking site in their N-terminal. Currently, only A and C type MAPKKs, such as AtMKK1, have been demonstrated to be activated by multiple abiotic stresses.^25^ Preivous studies have examined that MEKK1, MKK2, MPK4, and/or MPK6 respond to salt, drought, and cold stress in *Arabidopsis*.^26^ CsNMAPK regulated NO_3_^−^ stress,^27^ ROS, and osmotic adjustment under salt stress.^28^ Several reports revealed that MAPKKK play roles in drought resistance. A study in rice showed that DSM1, a RAF-Like MAPKKK gene has function in drought and oxidative stresses signaling.^29^ A tobacco MAPKKK (NPK1) has been shown to enhance drought tolerance in transgenic maize.^30^ The plant immune response is negatively controlled by the MEKK1(AtMEKK1)-MKK1(AtMKK1) and MKK2—MPK4-MKS1/WRKY33 cascades.^31,32^ AtMKK1-AtMPK6 are involved in ABA—induced CAT1 expression,^33^ MEKK1-MKK4/5 have an important function in plant innate immunity,^34^ and MKK6 can regulate cytokinesis.^35,36^

Studies on the evolution would be another interesting topic because their evolutionary history can shed light on functions. In land plants, the number of MAPKs and MAPKKs has expanded.^37,38^ Before the divergence of the rice and Brachypodium, MAPKKK genes experienced the segment duplication.^38^ The conservation of MAPKKKs have also implied by the strong purifying selection during the evolution.^39^ During the long evolutionary history, most of higher plant lineages have undergone polyploidization.^40^ In parallel with the whole-genome duplication (WGD) in angiosperm genomes, the three large families (MAPK, MAPKK and MAPKKK) experienced expansion and functional divergence. If the genes were involved in networks or function in a dose-sensitive manner, which should be retained that gene dosage hypothesis predicts.^41^ *Brassica rapa* has undergone two duplication events (WGD α and β) and one whole-genome triplication event (WGT γ) within the Brassicaceae lineage.^42^ Specifically, the WGT caused extensive fractionation in the *B. rapa* genome, which has the chance to study the molecular evolution of the MAPK cascade. Comparative analyses of many other plant species can yield the origin and evolution of these genes. Therefore, in addition to the detailed study of the MAPK cascade in *Brassica rapa,* we comparatively analyzed MAPK, MAPKK, and MAPKKK genes from eight representative plant species, including an algae (*Volvox carteri*), the ancestor of land plants,^43^ a bryophyte (*Physcomitrella patens*), the earliest sequenced land plant,^44^ a lycophyte (*Selaginella moellendorffii)*, an early vascular plant,^45^ and five angiosperm plants: *Amborella trichopoda,* one of the earliest angiosperm, *Vitis vinifera,* the basal eurosid, and *Populus trichocarpa, Carica papaya* and *A. thaliana*.

In this study, we comprehensively analyzed the MAPK cascade genes in the *B. rapa* genome, as well as representative genomes in the tree of plant life, to understand the cascade retention and expansion patterns following the WGT event. In addition, divergent tissue-specific expression patterns and response to stresses of these genes will help us to understand the function of the MAPK cascade genes.

## Materials and methods

### Retrieval of sequences

To identify MAPK cascade genes in *B. rapa*, *Arabidopsis*, and *O.sativa*, the sequences were retrieved from BRAD (http://brassicadb.org/brad/),^42^ TAIR (http://www.*arabidopsis*.org/) and RGAP (http://rice.plantbiology.msu.edu/),^46^ respectively. The sequences of others species were downloaded from Phytozome v9.1 (http://www.phytozome.net/).^47^ MAPK cascade genes of *Arabidopsis* were performed against the proteins from other species by BLASTP (*e*-value <1×10^−10^, identity >70%). The serine/threonine-protein kinase-like domain (PF00069), was downloaded from Pfam (http://pfam.sanger.ac.uk/), and used to search the *B. rapa* protein database by HMMER v3.0 (http://hmmer.janelia.org/). Potential sequences were detected by SMART (http://smart.embl-heidelberg.de/).

### Identification of gene synteny and duplicated MAPK cascade genes

According to previous reports, gene synteny and duplicated types were detected by the MCScanX (Multiple Collinearity Scan toolkit).^48^ The positions of syntenic MAPK cascade genes in the *B. rapa* on the ancestral genomic blocks were searched from the BRAD website (http://brassicadb.org/brad/searchSynteny.php).^42^ Circos software was used to draw syntenic diagram.^49^

### Phylogenetic analysis and characterization of the MAPK cascade genes

MEGA v6.0 was used to construct the maximum-likelihood (ML) trees with a bootstrap value of 1000 replications. MEGA v6.0 was used to calculate genetic distances. The MEMEv.4.10.1 tool (http://meme.sdsc.edu/meme/) was used to identify conserved motifs in the MAPK cascade proteins. The MAPK cascade gene structures were drawn by the online tool Gene Structure Display (GSDS, http://gsds.cbi.pku.edu.cn/). The STRING tool was used to construct interaction network of MAPK cascade proteins in *B. rapa* (http://string-db.org/).^50^

### The MAPK cascade genes Ka/Ks analysed

The MAPK cascade genes from *B. rapa* were aligned using ClustalX 2.0 with the default parameters.^51^ The KaKs_calculator was used to calculate the Ks and Ka values.^52^ The Ks values for all the MAPK cascade homologous genes in *B. rapa* and *A. thaliana* were shown by boxplot using the R program (boxplot package).^53^ The following formula: *T*=Ks/2*r* was used to calculate divergence time (*r*=of 1.5×10^−8^, synonymous substitutions per site per year).^54^ Nucleotide distance was used to analyze the relationships among Classes I, II, III and IV.

### Expression pattern analysis for MAPK cascade genes

Illumina RNA-seq data^55^ were used to characterize the expression of the MAPK cascade genes in *B. rapa*. The expression level of MAPK cascade genes were analysed by the fragments per kilobase of exon model per million mapped reads (FPKM). The AtGenExpress Visualization Tool (AVT; http://jsp.weigelworld.org/expviz/expviz.jsp) was used to analyse the expression level^56^ in *A. thaliana*. The R program (VennDiagram package) was used to draw Venn diagrams.

### Promoter sequence analysis

Search for *cis*-regulatory elements in promoter of MAPK cascade genes sequences was performed using the PLACE Web Signal ScanPLACE (http://bioinformatics.psb.ugent.be/webtools/plantcare/html/).

## Results

### Different retention of MAPK cascade genes following the WGT event in *Brassica rapa*

BLAST software was used to identify the number of MAPK, MAPKK, and MAPKKK genes in *A. thaliana* and *B. rapa*. Then, the MAPK cascade genes containing the serine/threonine-protein kinase-like domain was searched by an HMM (PF00069) in *B. rapa*. Finally, all the homogeneous candidate MAPK cascade sequences were subjected to Pfam, SMART and NCBI database analyses. After multiple screening and validation steps, we finally identified 34 BrMAPK, 14 BrMAPKK, and 112 BrMAPKKK genes (Supplementary Table S1).

**Figure 1:**
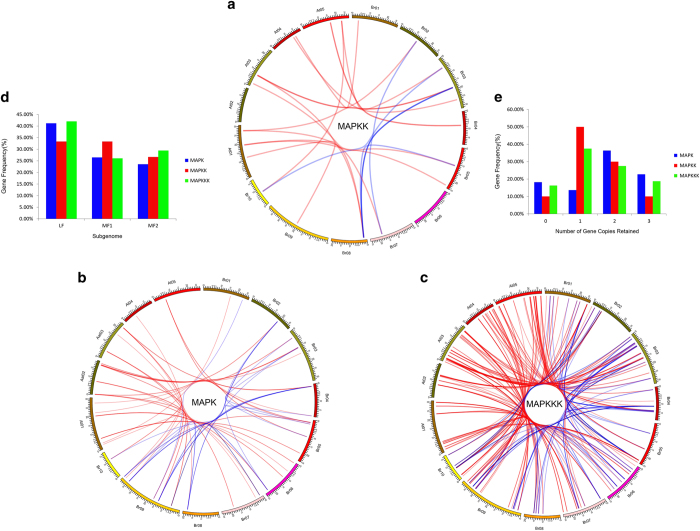
The syntenic MAPK cascade genes between *Brassica rapa* and *Arabidopsis thaliana* and their different retentions. (**a**–**c**) MAPK, MAPKK, MAPKKK syntenic genes lines are shown among the 10 *B. rapa* chromosomes (Br01-Br10) and the five *A. Thaliana* chromosomes (At01–At05). The red lines represent the syntenic genes pairs between *B. rapa* and *Arabidopsis*, and the blue lines represent the syntenic genes in *B. rapa*. (**d**) Retention of homoeologs of MAPK cascade genes in the three subgenomes of LF (least fractionized subgenome), MF1(moderately fractionized subgenome), and MF2 (most fractionized subgenome) in *B. rapa*. (**e**) Numbers of MAPK cascade genes after genome triplication and fractionation in *B. rapa*.

To investigate different syntenic genes in the MAPK cascade during the *B. rapa* WGT event, we completed a syntenic analysis with MCScanX. In total, 31 MAPKs, 14 MAPKKs, and 100 MAPKKKs were located in the syntenic regions (Figure 1). About 91% (31/34) of the MAPK genes, 93% (13/14) of the MAPKK genes, and 89% (100/112) of the MAPKKK were retained in the syntenic regions (Figure 1). We also counted the number of genes and analyzed the distribution of the three subgenomes by comparing the retention of MAPKs, MAPKKs and MAPKKKs (Figures 1d and e). The proportion of retained MAPK cascade genes was higher in the least fractionated (LF) subgenome than in the medium fractionated (MF1) and most fractionated (MF2) subgenome.This finding is consistent with a previous report in which the degree of retained genes in these three sub-genomes (LF, MF1, and MF2) decreased gradually.^42^ More MAPKKKs were retained in the MF2 sub-genome than in the MF1 sub-genome. MAPK (59%), MAPKK (40%), and MAPKKK (46%) genes retained more than two homologous genes, and the MAPK genes had higher retention than the two other gene families (MAPKK and MAPKKK). However, some MAPK (18%), MAPKK (10%) and MAPKKK (16%) genes were lost (Figure 1e). Therefore, MAPK genes were preferentially retained compared to the other two gene families. The MAPKK genes were similarly retained to MAPKKK genes following the WGT in *B. rapa* (Figure 1, Supplementary Table S2).

### Characteristic structure analysis of BrMAPK cascade proteins

**Figure 2:**
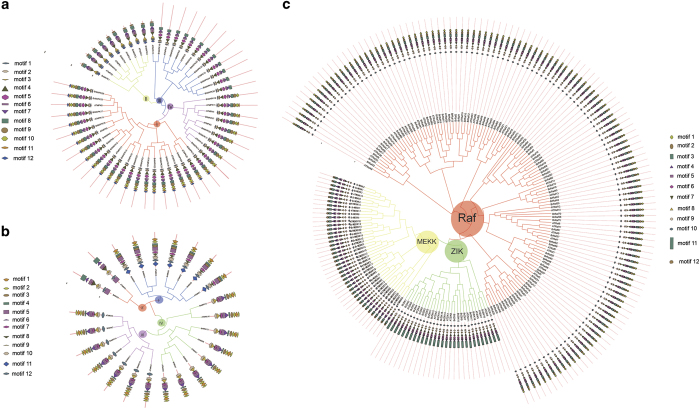
Schematic diagram of amino-acid motifs of MAPK cascade genes in *Arabidopsis* and *B. rapa*. (**a**) Phylogenetic relationships and protein motifs of MAPK genes. (**b**) Phylogenetic relationships and protein motifs of MAPKK genes. (**c**) Phylogenetic relationships and protein motifs of MAPKKK genes.

To study the characteristic of MAPK cascade genes in *B. rapa*, we used the Maximum Likelihood method to construct phylogenetic trees (Figure 2). All the BrMAPKs were classified into four classes, I, II, III and IV (Figure 2). We searched using the MEME tool to identify conserved motifs of BrMAPK proteins (Figure 2a). Eight of the 12 motifs were conserved in all the BrMAPK proteins, but the remaining 4 motifs (9–12) were not. In addition, each subfamily had its specific motif. For example, aside from the conserved motifs, MAPK proteins in classes I and II had specific motif 10 in their N-terminal region, whereas those in class IV contained motifs 8 and 9 in their C-terminal region (Figure 2). The annotated motifs from MEME revealed that eight of the 10 motifs (1–8) corresponded with the kinase domains in MAPKs (Supplementary Figure S1). Class II BrMAPK genes had no introns and all the BrMAPKK geenes contained a protein kinase domain (Supplementary Figure S1).

The BrMAPKK genes were clustered into four distinct gene classes (I, II, III and IV) (Figure 2b), and varied in kinase domain presence or absence, adenosine triphosphate (ATP) binding site and serine/threonine protein kinase active site according to phylogenetic analysis (Supplementary Figure S1). The BrMAPKK class III and IV subfamily genes have no introns, except for BrMAPKK9, which had one intron (Supplementary Figure S1). The BrMAPKKK gene family in *B. rapa* was clustered into three subfamilies (MEKK, RAF and ZIK) (Figure 2c). All the BrMAPKKK proteins also had a protein kinase domain (Supplementary Figure S1c).

### Duplication and Ks analysis for MAPK cascade genes in *A. thaliana* and *B. rapa*

We reconstructed 24 conserved ancestral genomic blocks (GBs) according to a previous report.^57^ The color-coding of these blocks was based on their positions in a proposed ancestral karyotype (AK1–8).^57,58^ To investigate the BrMAPK cascade genes of chromosomal location, all of them were mapped onto ten chromosomes in *B. rapa*, except *BrZIK4*, which is located on Scaffold000203 (Supplementary Figure S2). Among the ten sets of chromosomes in *B. rapa*, chromosome 09 contained more BrMAPK cascade genes (30 genes) than any of the other chromosomes. Among the LF, MF1 and MF2 subgenomes, LF contained more MAPK cascade genes than the MF1 and MF2 subgenomes. The most BrMAPKKK genes (18%) clustered in the AK8 region, whereas for BrMAPK and BrMAPKK genes, the most (20%) clustered in AK1 region (Supplementary Figure S2).

**Figure 3:**
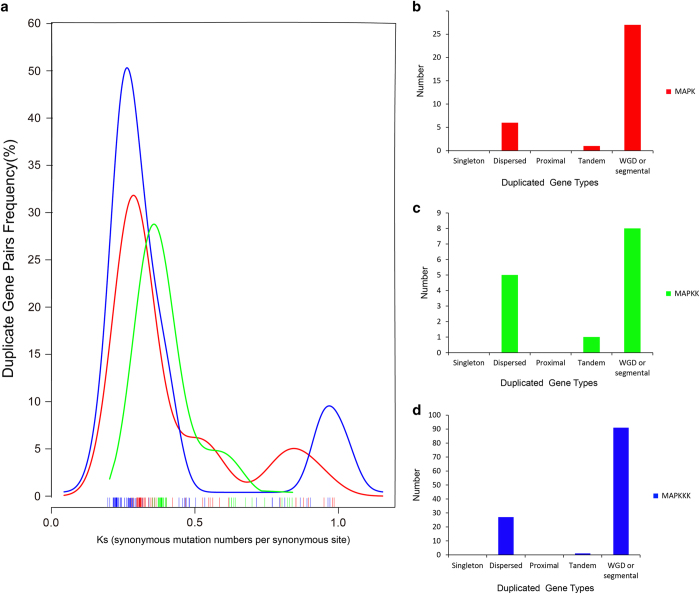
Pairwise comparison of the Ks values for MAPK cascade homologous genes in *B. rapa* and *A. thaliana* and their different duplicated types. (**a**) The distribution of Ks values for MAPK cascade genes between *B. rapa* and *A. thaliana*. (**b**–**d**) Singleton, dispersed, proximal, tandem and segmental duplicated types of MAPK cascade genes in *B. rapa*.

Gene duplication was the driving force for gene families evolution.^59^ We used the MCScanX software to detect the MAPK cascade genes duplication type. All of the BrMAPK cascade genes were traced to one of five duplication types (singleton, WGD, tandem, proximal or dispersed duplication types) (Figure 3). Twenty-seven BrMAPK, 8 BrMAPKK and 91 BrMAPKKK genes in *B. rapa* were duplicated from WGD or segmental events compared to only 1 BrMAPK, 1 BrMAPKK and 1 BrMAPKK genes from tandem duplication and 6 BrMAPK, 5 BrMAPKK and 27 BrMAPKK genes from dispersed duplication (Figure 3). The expansion of the BrMAPK cascade gene family was derived by the WGD or segmental duplication and dispersed gene duplication in *B. rapa*. The BrMAPKKK gene Ks values ranged from 0.2 to 0.5 and focused on about 0.25 (~8.5 Myr), whereas the BrMAPKK genes ranged from 0.4 to 0.7 and had a mean of about 0.40 (~12.5 Myr; Figure 3a and Supplementary Table S3). The BrMAPK gene Ks values ranged from 0.25 to 0.5 and centered ~0.3 (~10.5 Myr).The divergence time for the BrMAPKKK duplicated gene pairs was 8.49 MYA (Million years ago), which indicates that their divergence occurred during the *Brassica* triplication event (5–9 MYA). The BrMAPK and BrMAPKK duplicated gene happened 10 to 16.6 MYA and occurred during the divergence of *B. rapa* from *Arabidopsis* (9.6–16.1 MYA).

### Expansion and evolution of MAPK genes in plants

**Figure 4:**
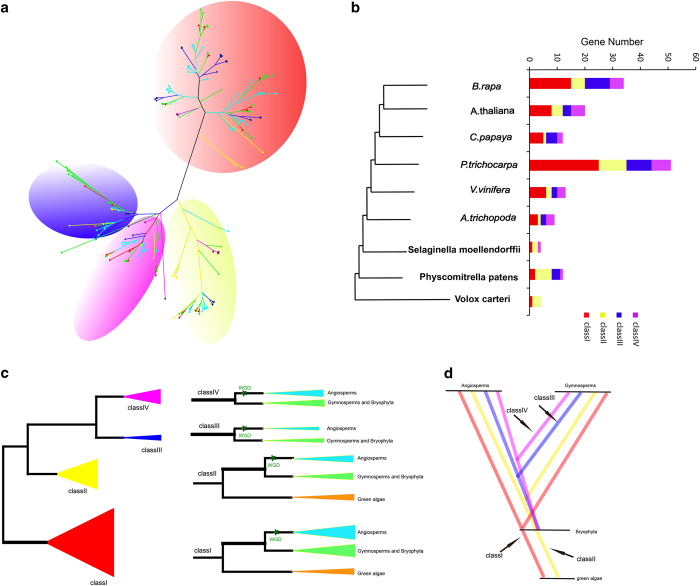
The duplication origin of MAPK genes in plants. (**a**) Phylogenetic relationships among MAPK genes. (**b**) Comparison of numbers of MAPK genes in representative species. (**c**) The phylogenetic relationships of MAPK genes. The green stars denote the inferred whole-genome duplication in angiosperms. (**d**) Schematic representation of the duplication history of MAPKs in plants. The red, yellow, blue and violet lines indicate class I, II, III and IV MAPK genes, respectively.

We performed a phylogenetic tree of MAPK genes with additional eight key species in the tree of plant life to survey the evolutionary relationships of the MAPK gene family in plants. We selected four angiosperms *Vitis vinifera, P. trichocarpa* and *C. papaya* because these species did not undergo α and β duplications, and *A. trichopoda* is a basal angiosperm that did not undergo the γ duplication event.^60–62^ A total of 204 MAPK genes were identified from the nine plant species (Supplementary Table S4; Figure 4a). All the MAPK genes segregated into four distinct classes (classes I, II, III and IV) with 100% bootstrap support (Supplementary Figure S3). The nucleotide distance between class I subfamily with class II, III and IV subfamilies was higher than those between MAPK classes II and III subfamilies, class II and IV subfamily, and classes III and IV subfamilies (Supplementary Figure S4). These findings indicated that class I had a distant phylogenetic relationship with the other three subfamilies, which is consistent with the results from the phylogenetic trees (Figure 4).

The gene numbers for the four classes in the MAPK gene family were marked in the nine plant species (Figure 4b). Only one class I and three class II subfamilies of MAPK genes were found in *Volvox carteri*, suggesting that MAPK gene expansion happened after the divergence of green algae. All these 9 species had conserved numbers (1–5) of class II MAPK genes, except *P. trichocarpa*, which had 10 MAPK genes. Based on the phylogenetic relationships, the class II subfamily of MAPK genes originated from green algae, whereas classes III and IV subfamilies originated from land plants (Figure 4c). Class II subfamily of MAPK genes diverged from Bryophyta class III and IV subfamilies, and that the rapid expansion of classes II, III and IV subfamilies may have primarily led to MAPK gene expansion in angiosperm plants. The evolution of MAPK genes was re-constructed and suggests that classes III and IV subfamilies originated from the class II subfamily, which was the initial group, and that the class III subfamily separated from the class IV subfamily in plants classified in the Bryophyta (Figure 4d).

### Expansion and evolution of MAPKK genes in plants

**Figure 5:**
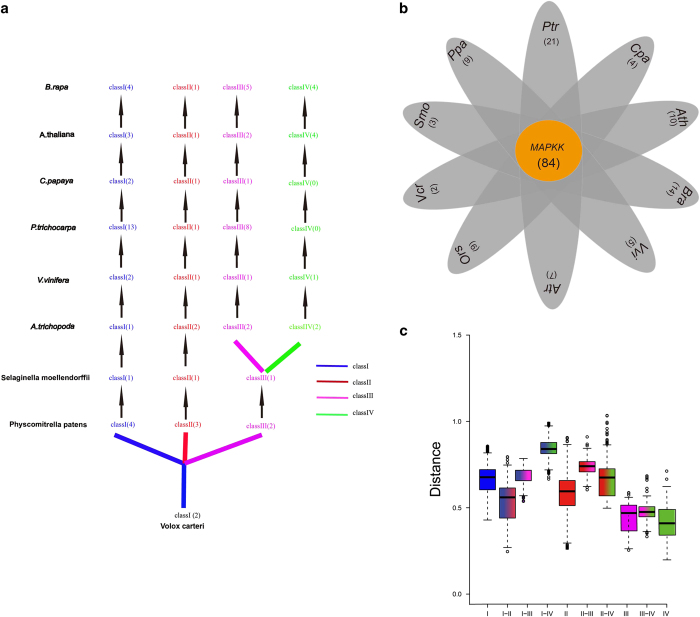
The analysis of MAPKK gene evolution. (**a**) Comparison of numbers of MAPKK genes in the phylogenetic tree representing plant species. (**b**) Venn diagram shows the number of common gene families and genes in 10 plants. (**c**) Genetic distance among the different classes of MAPKK genes.

Based on the phylogenetic trees for all the MAPKKs, we tried to re-construct the expansion history of the MAPKK families. A total of 84 MAPKK genes were identified in the nine species, although only two MAPKK genes were detected in *V. carteri*. According to the phylogenetic tree topologies, the MAPKK genes could be grouped into four classes (classes I–IV) (Supplementary Figure S5). The MAPKK genes from *P. patens* and *S. moellendorffii* were clustered into only three MAPKK subfamilies included classes I, II and III, whereas all MAPKK subfamilies were present in the angiosperms, which expanded rapidly, and was accompanied by several WGD (Supplementary Figure S6). The number of MAPKK genes in most angiosperms was greater than that in the other non-angiosperm species (*V. carteri* and *P. patens*), and was most likely due to WGDs, which leads to gene expansion (Figure 5). The number of MAPKK genes in class II was a highly conserved in the all species except *A. trichopoda* (2), *P. pteridophyta* (3) and *V. carteri* (no class II MAPKK genes were identified; Figures 5a and b). One class I MAPKK gene subfamily was found in *V. carteri* and suggested that MAPKK gene expansion occurred in land plants. However, no class II MAPKK genes were detected in *V. carteri* that indicated the class II gene subfamily may be unique to land plants.

The four MAPKK classes have similar domain architectures and motif compositions (Supplementary Figure S7). In addition to the domain compositions identified by pfam, 10 common motifs embedded in MAPKK gene domains were identified by MEME for all of the MAPKK genes. Class I subfamily of MAPKK genes had motif 8. Although the two MAPKK genes in *V. carteri* do not have motif 8, all the other species have similar motif compositions. From algae to angiosperm, the MAPKK gene family has highly conserved domains and motifs.

Phylogenetic trees were constructed in the same nine species to investigate the evolution of the MAPKK genes (Supplementary Figure S6). Genetic distances were analyzed to examine the relationships among the four classes (Figure 5c). The genetic distances for class III subfamily versus class IV subfamily were smaller than class III versus class II subfamily, and class III subfamily versus class I subfamily, which indicated that class III subfamily had a closer relationship with class IV subfamily. Moreover, the genetic distances for classes I, II, and III subfamilies were similar, whereas that of subfamily IV was the smallest, which suggested that the degree of sequence divergence for class IV subfamily was lower than the other subfamilies (Figure 5c). The evolutionary history of MAPKKs was surmised that class I subfamily was the initial class from which MAPKKs originated. Additionally, the class IV subfamily separated from class III subfamily in angiosperm plants.

### Expansion and evolution of MAPKKK genes in plants

**Figure 6:**
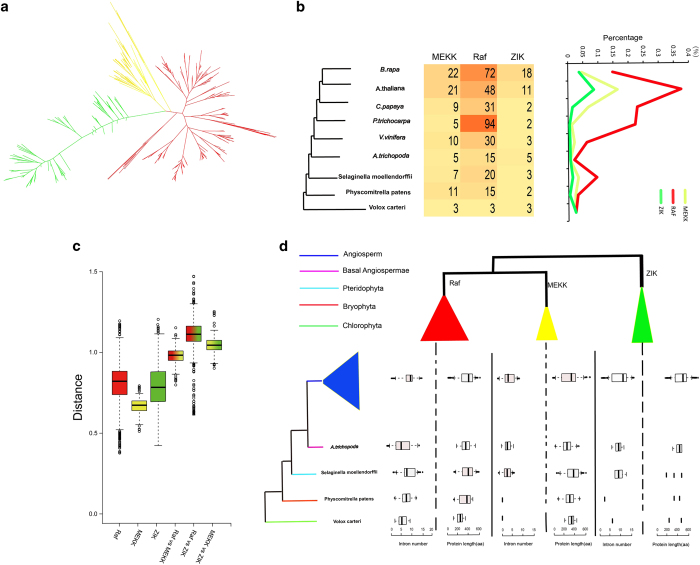
The analysis of MAPKKK genes evolution. (**a**) Phylogenetic relationships among MAPKKK genes. (**b**) The percentage and numbers of MAPKKK genes in representative species. (**c**) Genetic distance among the different clades of MAPKKK genes. (**d**) Distribution of structural characteristics of MAPKKK genes in plant kingdom.

We constructed a phylogenetic tree of the MAPKKK genes that clustered into three subfamilies (MEKK, ZIK and Raf) (Figure 6a, Supplementary Figure S8). The MEKK, ZIK and Raf subfamily were detected in *V. carteri* (Figure 6b) that suggested they all originated from the green algae. Genetic distances were analyzed to further determine the relationships among the three subfamilies (Figure 6c). The genetic distances between MKKK subfamily and the Raf subfamily were smaller than MEKK subfamily versus other subfamilies (Figure 6c). These results indicated that MEKK subfamily has a close relationship with Raf subfamily and that MEKK and Raf subfamilies may share a common evolutionary origin.

Analysis of MAPKKKs family size in nine plants indicated that it underwent rapid expansion during evolution, and further expansion in the Brassicaceae (Figure 6b). Phylogenetic trees were constructed for each species and the MAPKKK family divided into three classes (MEKK, ZIK and Raf) (Supplementary Figure S8). The WGD had important role in the expansion and evolution of gene families in plant genomes, the rapidly increased gene numbers of the MAPKKK family in *B. rapa* (18) and *A. thaliana* (11) (Figure 6b) gave a good example for that. Notably, the number of MAPKKKs in *P. trichocarpa* and *B. rapa* is more than other species, which was caused by salicoid duplication and Brassica-specific WGD events. During expansion of the MAPKKK gene family, the Raf subfamily played a major role in this process.

We compared gene structure characteristics between algae and higher plants to better understand the evolutionary characteristics of MAPKKK genes in the plant kingdom (Figure 6d). The gene structure of Raf was quite simple in lower species with most genes having just five introns, whereas the number of introns increased to about 10 in the angiosperms (Figure 6d). The length of the protein has been stable at about 386 amino acids. MEKKs have no introns in *V. carteri* and *P. pteridophyta* (Figure 6d).

### Comparative expression patterns of the MAPK cascade genes in different tissues from *B. rapa* and *A. thaliana*

**Figure 7:**
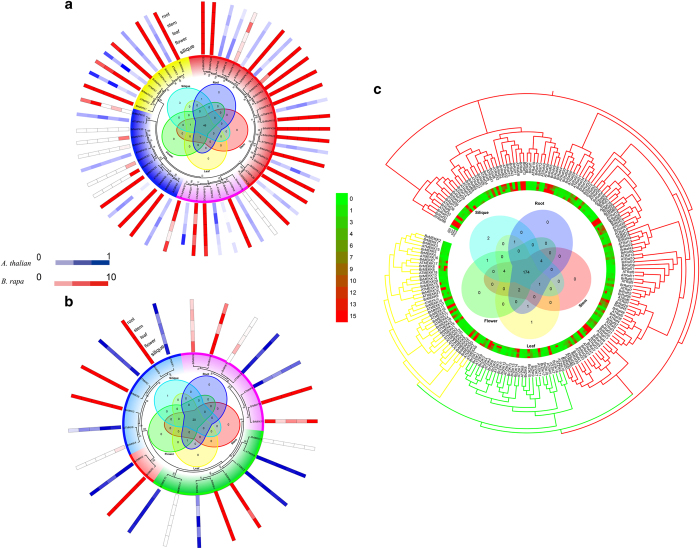
Expression pattern analysis of MAPK cascade genes in *A. thaliana* and *B. rapa.* Heat map representation and hierarchical clustering of MAPK cascade genes ((**a**) MAPK, (**b**) MAPKK, (**c**) MAPKKK) in root, stem, leaf, flower and silique. Venn diagram depicting the distribution of shared expression of the MAPK cascade genes among five *Brassica rapa* tissues.

The MAPK cascade gene expression patterns between *A. thaliana* and *B. rapa* were investigated in roots, stems, leaves, flowers and siliques (callus only was studied in *B. rapa* and mature pollen studied in *A. thaliana*; Figure 7 and Supplementary Tables S5 and S6). Gene expression of BrMAPKs was detected in the roots, stems, leaves and flowers (28 BrMAPKs, 82%) and siliques (31BrMAPKs, 91%; Figure 7a). All AtMAPKs showed expression (mean—normalized value >1) in all tissues (Figure 7a). Gene expression of BrMAPKK and AtMAPKK was detected in at least one of the tissues, except *BrMAPKK13* and *BrMAPKK14* in which no expression was found in any of the tissues. Among the 196 BrMAPKKKs (including 84 AtMAPKKKs and 112 BrMAPKKKs), 5 (*BrRaf36*, *BrRaf70*, *BrRaf63*, *BrZIK11*, and *BrRaf71*) were not expressed, and 2 (*BrMEKK7* and *BrRaf21*) were expressed only in the flower tissue (Figure 7c and Supplementary Figure S9). Additionally, a total of 174 MAPKKKs had high expression levels (FPKM value >10) in the five tissues. We also analyzed MAPK cascade genes for tissue-specific expression profiles in five tissues; some BrMAPK cascade genes were highly expressed (FPKM value >10) in flowers, including *BrMAPK32*, *BrMAPKK12,* and *BrMEKK7*, whereas they had no or low expression in the other tissues (Figures 7a and c).

MAPK cascade genes in the five tissues on the phylogenetic tree were selected to survey divergent functions of the homologous genes (Figure 7). AtMAPKKs Class IV subfamily showed high expression (mean-normalized value >1) in the five tissues, except *BrMAPKK13* and *BrMAPKK14* were not expressed. Class III AtMAPKs also showed expression in all five tissues, except *BrMAPK30* and *BrMAPK34* were not expressed, and *BrMAPK31*, *BrMAPK32* and *BrMAPK33* were expressed only in the roots. These results indicated that class IV MAPKK and class III MAPK genes may have lost some of their functions after duplication events according to the expression patterns.

### *Cis*-elements and interaction network analysis among MAPK cascade proteins in *B. rapa*

**Figure 8:**
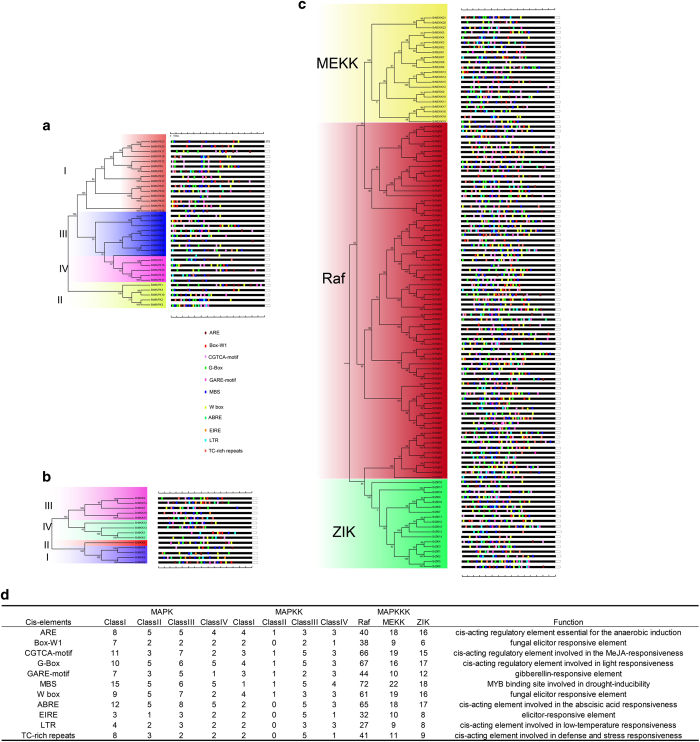
The *cis*-elements of the promoter regions of *B. rapa* MAPK cascade genes. The micro-segments in different colors were the putative elements sequences. The description of the ten *cis*- elements are in brackets.

To analyze the *cis*-regulatory elements, we used the Plant *Cis*-acting Regulatory DNA Elements (PLACE) online tool to identify MAPK cascade genes in *B. rapa*. A total of 11 common *cis*-regulatory elements were detected in the promoter regions of the MAPK cascade genes and were highly conserved among all of the MAPK cascade genes studied in *B. rapa* (Figure 8). Three common *cis*-regulatory elements, ARBE, the CGTCA-motif, and the GARE-motif, were responsive to plant hormones, including ABA, JA and GA, and suggested that they could affect MAPK cascade gene expression levels in *B. rapa*. Some common *cis*-regulatory elements responsive to both abiotic and biotic stresses were fungal elicitor-responsive elements (W-box and Box-W1), a light-responsive element (G-Box), low-temperature responsiveness (LTR), defense and stress responsiveness (TC-rich repeats), and a drought-responsive element (MBS). The diverse regulatory elements indicated the importance of MAPK cascade genes in stress tolerance. There were two more elements including a regulatory element essential for anaerobic induction (ARE) and an elicitor-responsive element (EIRE). In addition, we statistics the number of genes that contained *cis*-regulatory in each subfamily (Figure 8d). In the MAPK, most genes contianed more MBS *cis*-regulatory elements than other *cis*-regulatory elements. Only the class II subfamily of MAPKK had six *cis*-regulatory elements. In MAPKKK, most genes also contained *cis*-regulatory elements of MBS, which is similar to MAPK genes. These results indicated that MAPK cascade genes were responsive to various stresses, which may be due to the binding of corresponding *cis*-elements that regulate the expression of MAPK cascade genes.

The interaction network of BrMAPK cascade proteins was constructed by STRING software (Supplementary Figure S10). The lines stand for the positive correlations that were retrieved from STRING database corresponding to the protein interactions. The interaction netwok contained 42 nodes and 167 pairs of interacting genes. It showed a very complicated correlation among the BrMAPK cascade proteins (Supplementary Figure S10). This phenomenon indicated that MAPK cascade genes are involved in many fundamental mechanisms and regulate many downstream factors and/or are regulated by many upstream genes.

## Discussion

MAPK cascade genes have been shown to be involved in growth, development and responses to a variety of stress stimuli. The MAPK signaling pathway is one of the most important and conserved ubiquitous modules for signal transduction in plants ^14,20,21,63,64^ (Supplementary Figure S11).

If genes products are involved in complex regulatory networks, via different metabolic pathways or transcriptional regulation, they are likely to be retained.^41,65^ In this study, we identified 34 BrMAPK, 15BrMAPKK and 112 BrMAPKKK genes in the *B. rapa* genome that displayed high retention after the WGD. More MAPK (59%) and MAPKKK (46%) genes retained two or three copies in *B. rapa* than MAPKK (40%) genes. By analyzing the duplicated types, we found more segmental duplication in BrMAPKs (79.4%) and BrMAPKKKs (81.3%) than in BrMAPKKs (53.3%). BrMAPKs and BrMAPKKKs that participate in development, stress and defense, and play particularly important roles in signaling pathways. This result is agreement with the gene dosage hypothesis.^66^ Comparison of nucleotide distances showed that BrMAPKs and BrMAPKKKs diverged approximately 8.5 MYA, earlier than BrMAPKKs that separated at 12.5 MYA.

During evolutionary, angiosperm genomes have experienced polyploidization events, which have led to the MAPK cascade gene expansion, followed by divergence by (1) subfunctionalization, (2) neofunctionalization, or (3) nonfunctionalization.^62,67–69^ These fates have provided choices for duplicated genes to gain functional diversification.

We determined the evolutionary histories of the MAPK cascade genes through comparison analyses that examined expansion, phylogenetic relationships, characteristic structures and nucleotide distances. The MAPK class II subfamily had a close relationship with the classes III and IV subfamilies. We constructed a MAPK evolutionary model whereby classes III and IV subfamilies might have originated from class II, which was the ancestral group, followed by class III subfamily separating from class IV subfamily in Bryophyta (Figure 4). At present, MAPK cascade gene family members have been reported from a few plant species, including rice (*Oryza sativa*),^70^ Arabidopsis (*A. thaliana*),^71^ maize (*Zea mays*),^72^ Brachypodium distachyon,^73^ canola (*B. napus*),^74^ apple (*Malus domestica*)^75^ and grape (*V. vinifera*).^76^ MAPK genes had conserved amino acids sequences that reflects their common evolutionary ancestry.^77^ Before the divergence of the monocots and eudicots, the plant MPK gene family structure already was established from an ancient pattern of diversification.^77^ MAPK cascades play central roles in the eukaryotic signal transduction networks, and they have highly conserved component kinases during evolution.^78^

In flowering plants, MAPK genes number was twice that of MAPKKs.^37^ The MAPKK gene family has retained highly conserved domains and motifs from algae to angiosperms (Supplementary Figure S7). In their evolutionary, class II and III subfamilies of MAPKK genes might have originated from class I subfamily, which was the ancestral class, followed by class IV subfamily separating from class III subfamily in angiosperms. The MAPKK genes in *Arabidopsis* and *B. rapa* possessed similar gene complements and experienced several recent gene duplication events. After comparing the MAPKK genes in *V. vinifera* and *B. rapa*, we found that *B. rapa* had a relatively large number of MAPKK genes compared to *V. vinifera*. The reason might be that *V. vinifera* had not undergone α and β duplication events. The MAPKK gene family formed four distinct subfamilies in the phylogenetic tree, which is consistent with the result for *B. rapa* and *A. thaliana*.^37^

**Figure 9:**
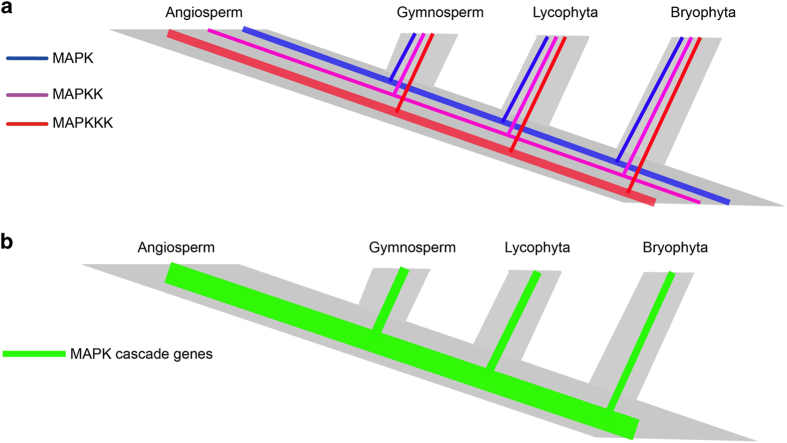
Evolutionary history of the MAPK cascade genes in the plant kingdom.

In the three subfamilies in the MAPKKK family, MEKK has a closer relationship to Raf than MEKK to ZIK, and plant MEKKs and Rafs may share a common evolutionary origin. Moreover, the increase of Rafs played a major role in the expansion of the MAPKKK gene family. An evolutionary history of MAPK cascade gene family was inferred in the plant kingdom; all three gene families exist in the genomes from Bryophyta to Angiospermae (Figure 9, Supplementary Figure S12).

Many of the MAPK pathway members have been characterized according to their response to various environmental stimuli in plants.^63^ The MEKK1-MPK4 cascade plays an important role in ROS (reactive oxygen species) metabolism,^79^ and MKK1–MPK6 cascade is involved in H_2_O_2_ metabolism.^33^ A TIPK (*Trichoderma*-induced MAPK) was reported to response to pathogen,^80^ whereas CsNMAPK regulates NO_3_^−^ stress,^27^ and salt stress.^28^ Plant MAPKs play roles in different hormones pathways,^81^ such as MKK3/MPK6 participate in jasmonic acid (JA) signaling.^82^ MAPKs were involved in abscisic acid (ABA) signaling pathway.^33^ To better understand the possible transcriptional regulation functions of MAPK cascade genes in *B. rapa*, we identified common conserved *cis*-regulatory elements in the promoter regions of MAPK cascade genes. We also found that 11 common *cis*-elements in all the MAPK cascade genes of *B. rapa* were responsive to plant hormones and environmental stresses.

## Conclusions

In summary, genome-wide analysis of MAPK cascade gene families was first performed in *B. rapa*, and 34 BrMAPK, 14 BrMAPKK and 112 BrMAPKKK genes were identified. We analyzed the characteristic structures, evolutionary patterns, gene duplication, expression divergence and *cis*-regulatory elements of MAPK cascade genes in plants. Comparative evolutionary analysis from eight other plants was also conducted in the tree of plant life. Our study provides new insights into the evolution of the MAPK cascade gene family in *B. rapa* and the plant kingdom in general. We further confirmed the significant regulation of the MAPK cascade genes in *B. rapa* during growth, development and stress tolerance. Our study provides useful information regarding the MAPK cascade genes functional divergence and conservation, and also aids in understanding the effects of polyploidy during evolution with regards to MAPK cascade genes.
