# Supplementary material for: Divergent evolutionary patterns of the MAPK cascade genes in Brassica rapa and plant phylogenetics
Source: Hortic Res. 2017 Dec 27;4:17079–. doi: 10.1038/hortres.2017.79 (PMC5744264; doi:10.1038/hortres.2017.79)
Supplement: Supplementary Information [file hortres201779-s1.doc]

**Divergent evolutionary patterns of the** **MAPK cascade genes** **in *Brassica rapa* and plant phylogenetics**

**Short title:** **Evolutionary patterns of MAPK cascade genes in *Brassica rapa***

**Peng Wu^1^, Wenli Wang^1^, Ying Li^1^, Xilin Hou^1*^**

^1^State Key Laboratory of Crop Genetics and Germplasm Enhancement/Key Laboratory of Biology and Germplasm Enhancement of Horticultural Crops in East China, Ministry of Agriculture, College of horticulture, Nanjing Agricultural University, Nanjing 210095, China;

***Corresponding author**: Xilin Hou; College of Horticulture, Nanjing Agricultural University, Nanjing, China.

Tel: +86 025 8439 5917;

Fax: +86 025 8439 5262; Email: hxl@njau.edu.cn.

**Figure S1.** Schematic diagram of protein domain and gene structures of MAPK cascade genes in *B. rapa*.

**Figure S2.** The distribution of MAPK cascade genes on ten chromosomes. The 8 ancestral blocks and three subgenomes, including the least fractionated (LF), medium fractionated (MF1), and most fractionated (MF2) subgenomes, were plotted as described by Cheng et al.(2013). AK represents the ancestral karyotype

**Figure S3.** Information of MAPK gene family clades in *Brassica rapa*, *Arabidopsis thaliana*, *Carica papaya*, *Populus trichocarpa*, *Vitis vinifera,* *Amborella trichopoda ,S. moellendorffii* ,*P. patens* and *V. carteri*. The α, β, γ, and salicoid duplications and the Brassica-specific triplication are indicated on the branches of the trees according to the Plant Genome Duplication Database. To classify these *MAPK* genes, phylogenetic trees with *MAPK* genes for each of the nine species by maximum likelihood using MEGA6.

**Figure S4.** The genetic distance among the different classes of MAPK genes.

**Figure S5.** The phylogenetic trees with *MAPKK* genes in *Brassica rapa*, *Arabidopsis thaliana*, *Carica papaya*, *Populus trichocarpa*, *Vitis vinifera,* *Amborella trichopoda ,S. moellendorffii* ,*P. patens* and *V. carteri*, respectively.

**Figure S6.** The phylogenetic trees of *MAPK K* genes in nine plants.

**Figure S7.**Schematic diagram of amino acid motifs of *MAPK K* genes in nine plants.

**Figure S8.** The phylogenetic trees with *MAPKKK* genes in *Brassica rapa*, *Arabidopsis thaliana*, *Carica papaya*, *Populus trichocarpa*, *Vitis vinifera,* *Amborella trichopoda ,S. moellendorffii* ,*P. patens* and *V. carteri*, respectively.

**Figure S9.** Venn diagram depicting the distribution of shared expression *MAPKKK* genes among six *Arabidopsis thaliana* tissues, i.e.root, leaf, silique, steam, flower and mature pollen

**Figure S10.** Interaction network of MAPK cascade genes in *B.rapa*

**Figure S11.** Possible functions of various mitogen-activated protein kinase (MAPK) cascades in plants. Homologous components of the various MAPK cascades are shown in the same color. Arrows indicate activation and bars indicate inhibition. Question marks represent unidentified components in the pathways. MAPKs that belong to different MAPK subfamilies are activated by various endogenous and exogenous stimuli in plants. The sharing of a common component between different cascades could create cross-talk between different pathways. Abbreviations: ABA, abscisic acid; HR, hypersensitive response; JA, jasmonic acid; MAPKK, MAPK kinase; MAPKKK, MAPKK kinase; SA, salicylic acid; SAR, systemic acquired resistance.

**Figure S12.** **The phylogenetic trees with MAPK cascade genes in plant (*Brassica rapa, Arabidopsis thaliana, Carica papaya, Populus trichocarpa, Vitis vinifera, Amborella trichopoda ,S. moellendorffii ,P. patens and V. carteri*).**
