# Supplementary figures and images for: Divergent evolutionary patterns of the MAPK cascade genes in Brassica rapa and plant phylogenetics
Source: Hortic Res. 2017 Dec 27;4:17079–. doi: 10.1038/hortres.2017.79 (PMC5744264; doi:10.1038/hortres.2017.79)

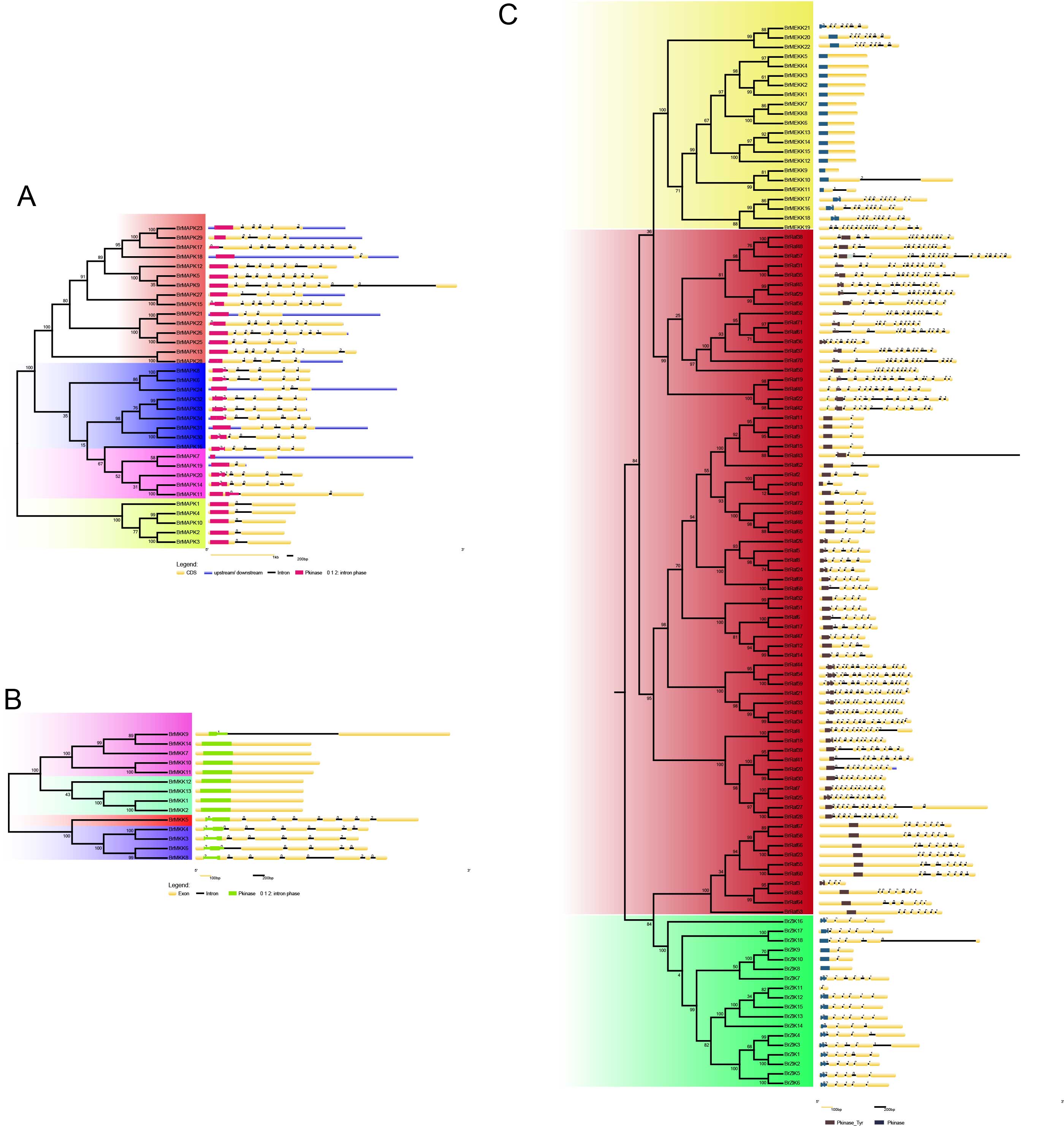

Supplement: Supplementary Figure S1 [file hortres201779-s3.jpg]

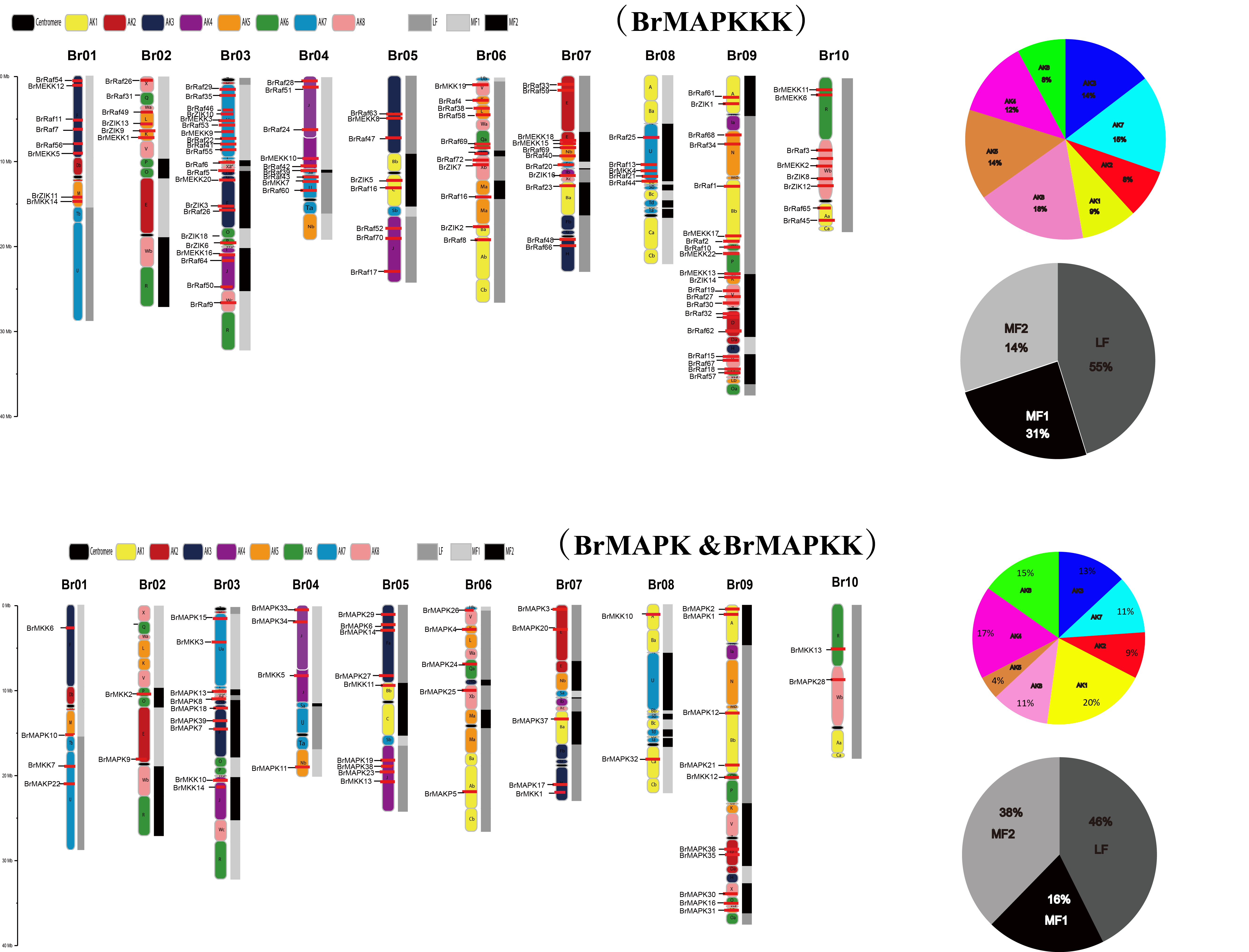

Supplement: Supplementary Figure S2 [file hortres201779-s4.jpg]

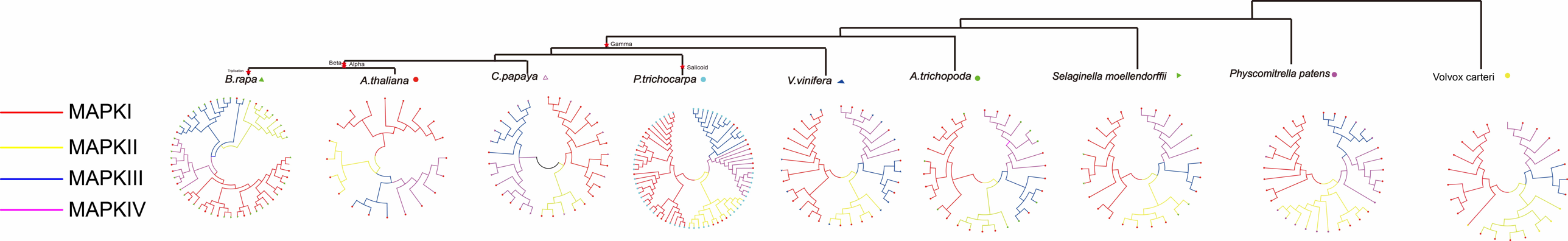

Supplement: Supplementary Figure S3 [file hortres201779-s5.jpg]

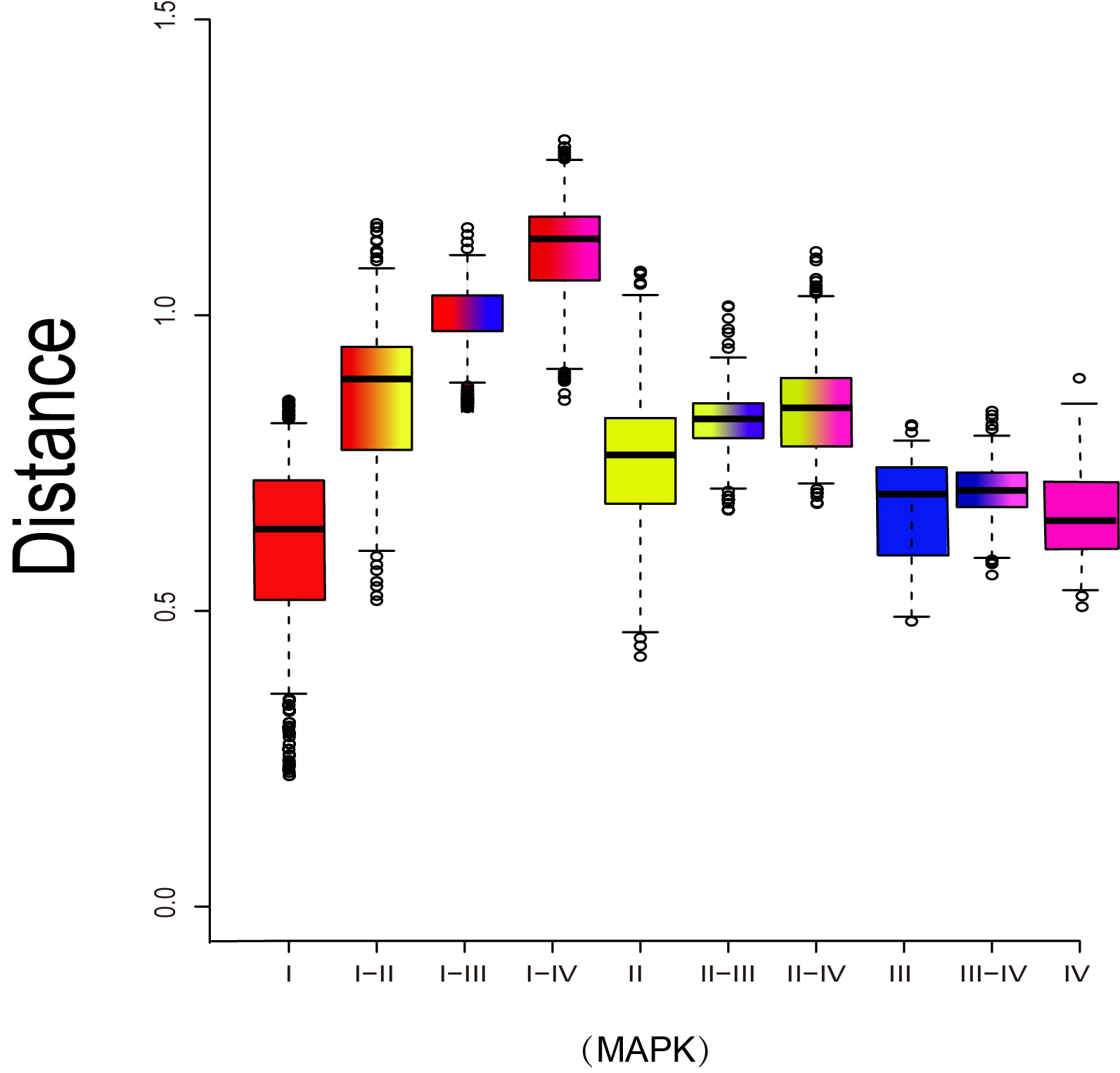

Supplement: Supplementary Figure S4 [file hortres201779-s6.jpg]

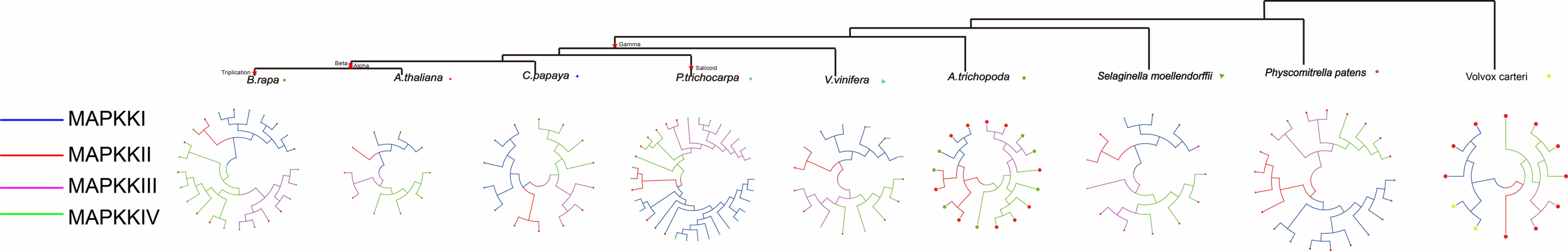

Supplement: Supplementary Figure S5 [file hortres201779-s7.jpg]

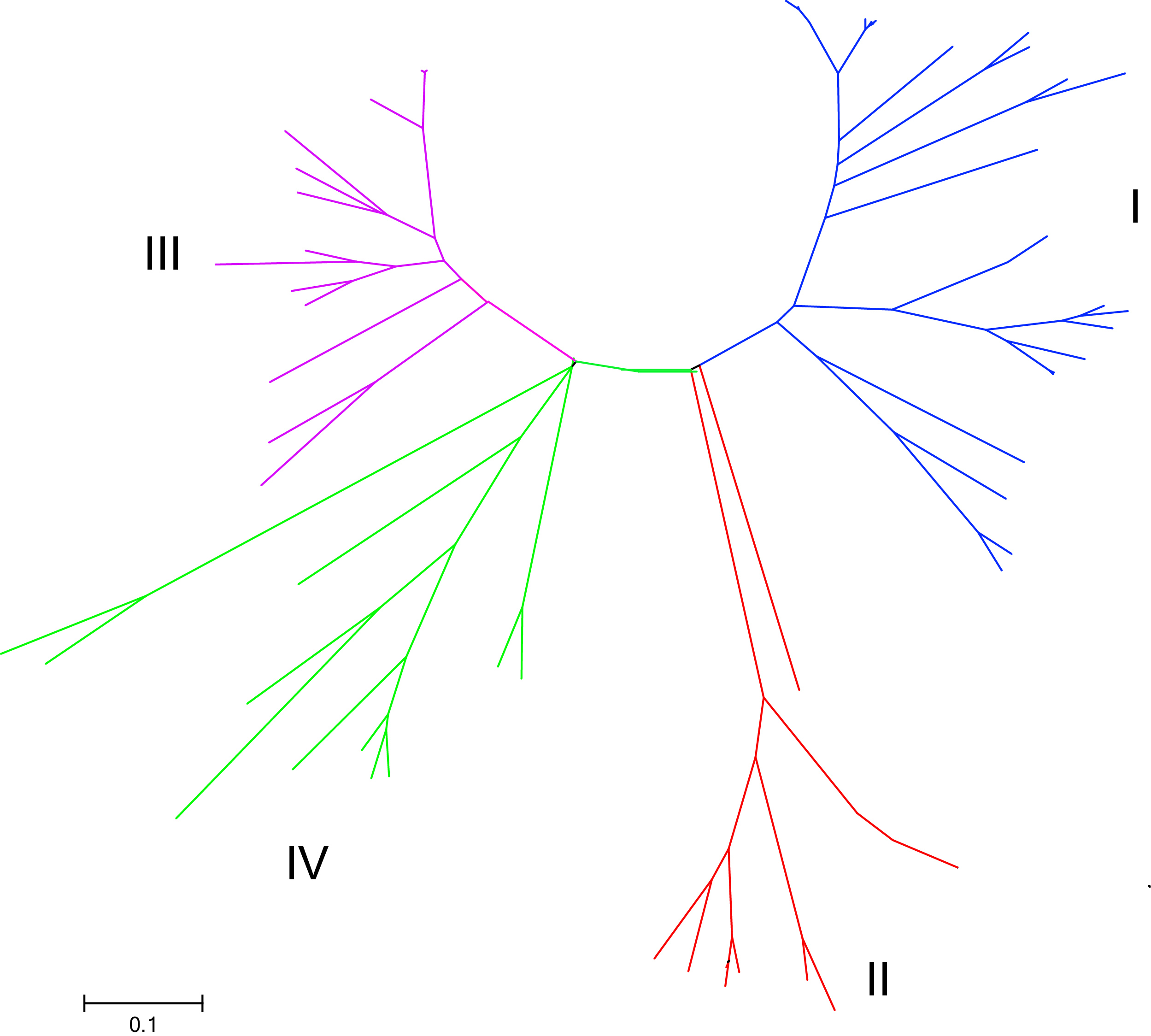

Supplement: Supplementary Figure S6 [file hortres201779-s8.jpg]

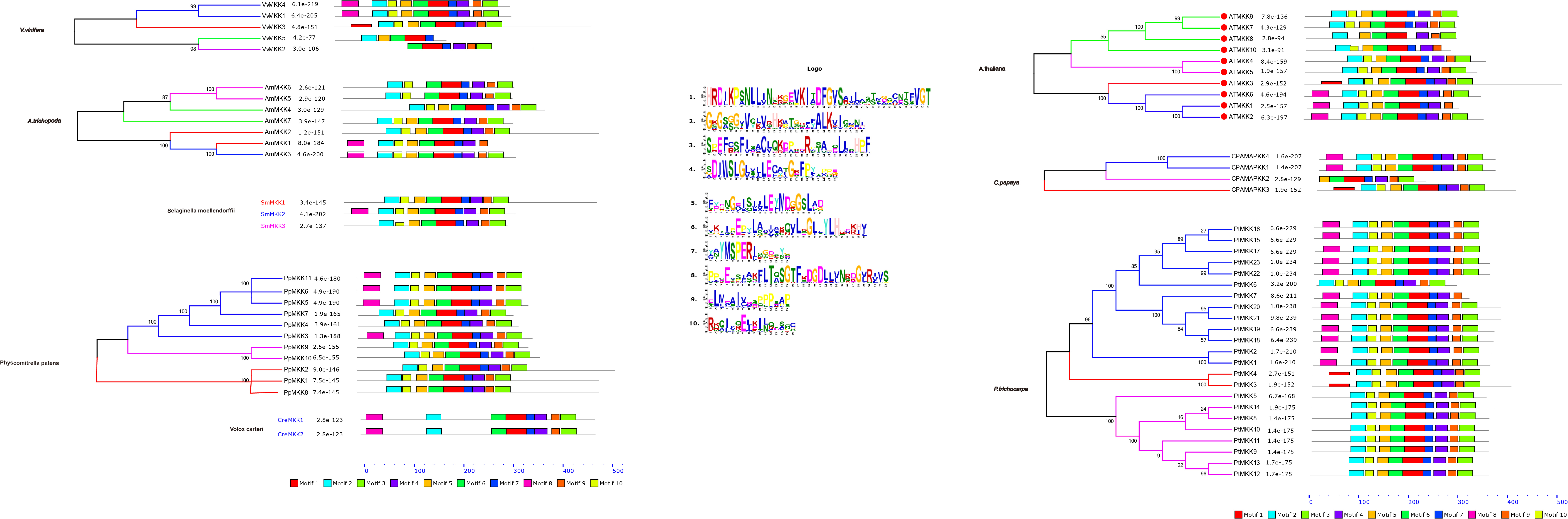

Supplement: Supplementary Figure S7 [file hortres201779-s9.jpg]

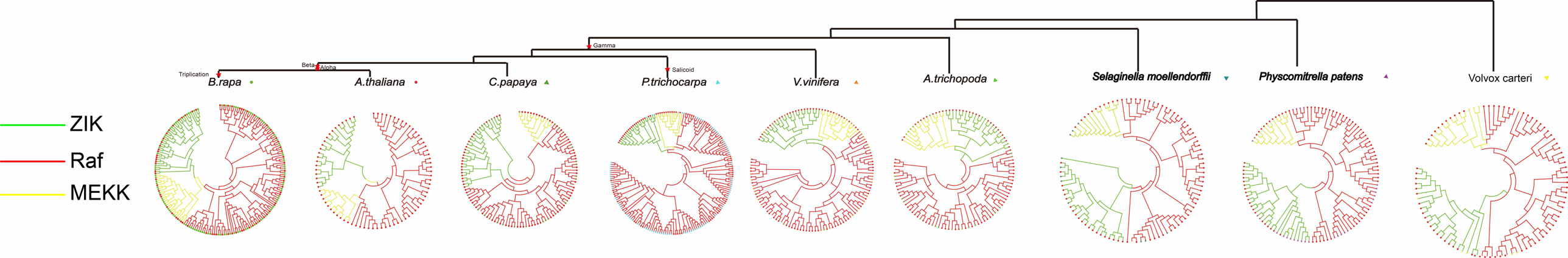

Supplement: Supplementary Figure S8 [file hortres201779-s10.jpg]

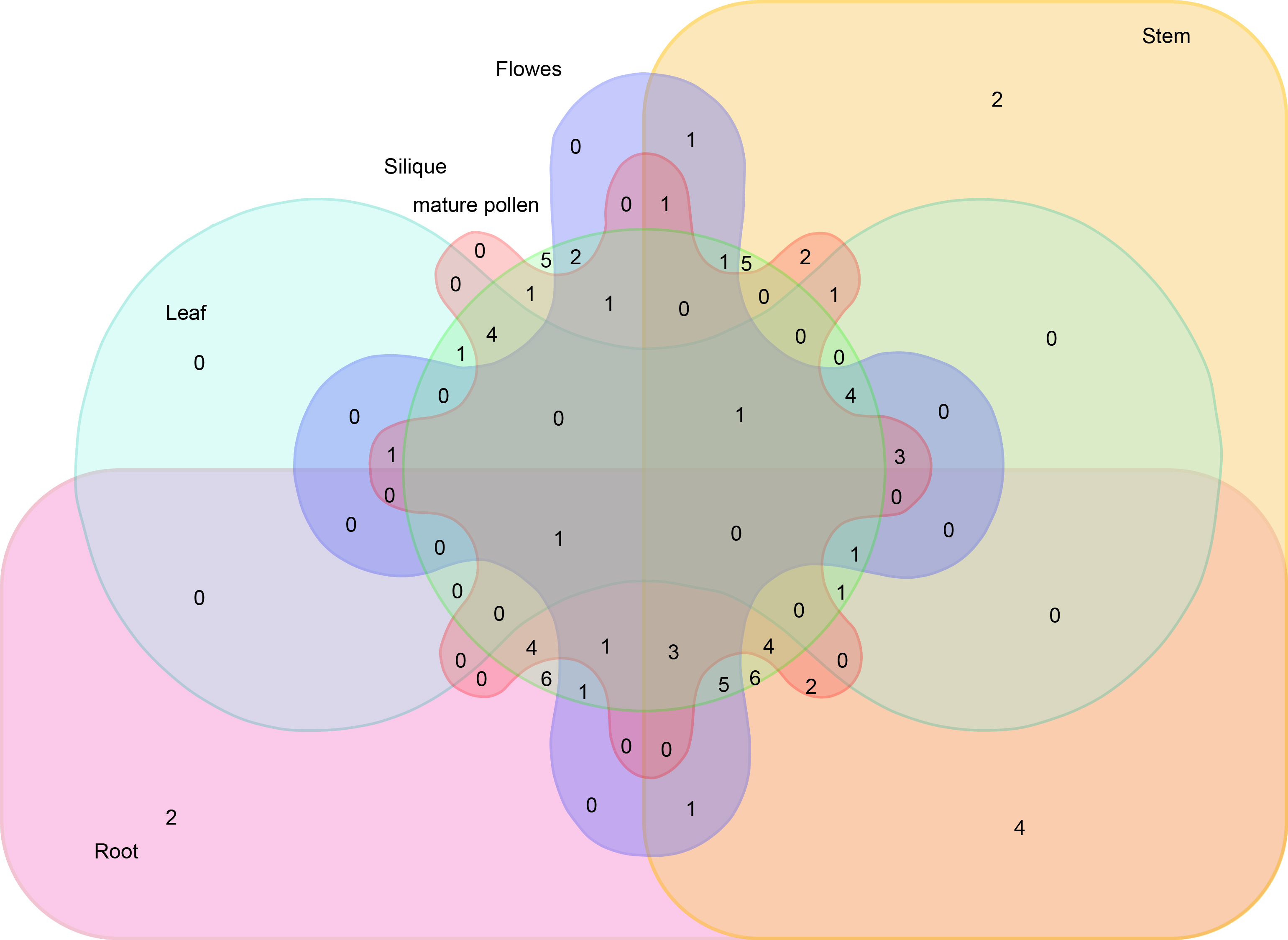

Supplement: Supplementary Figure S9 [file hortres201779-s11.jpg]

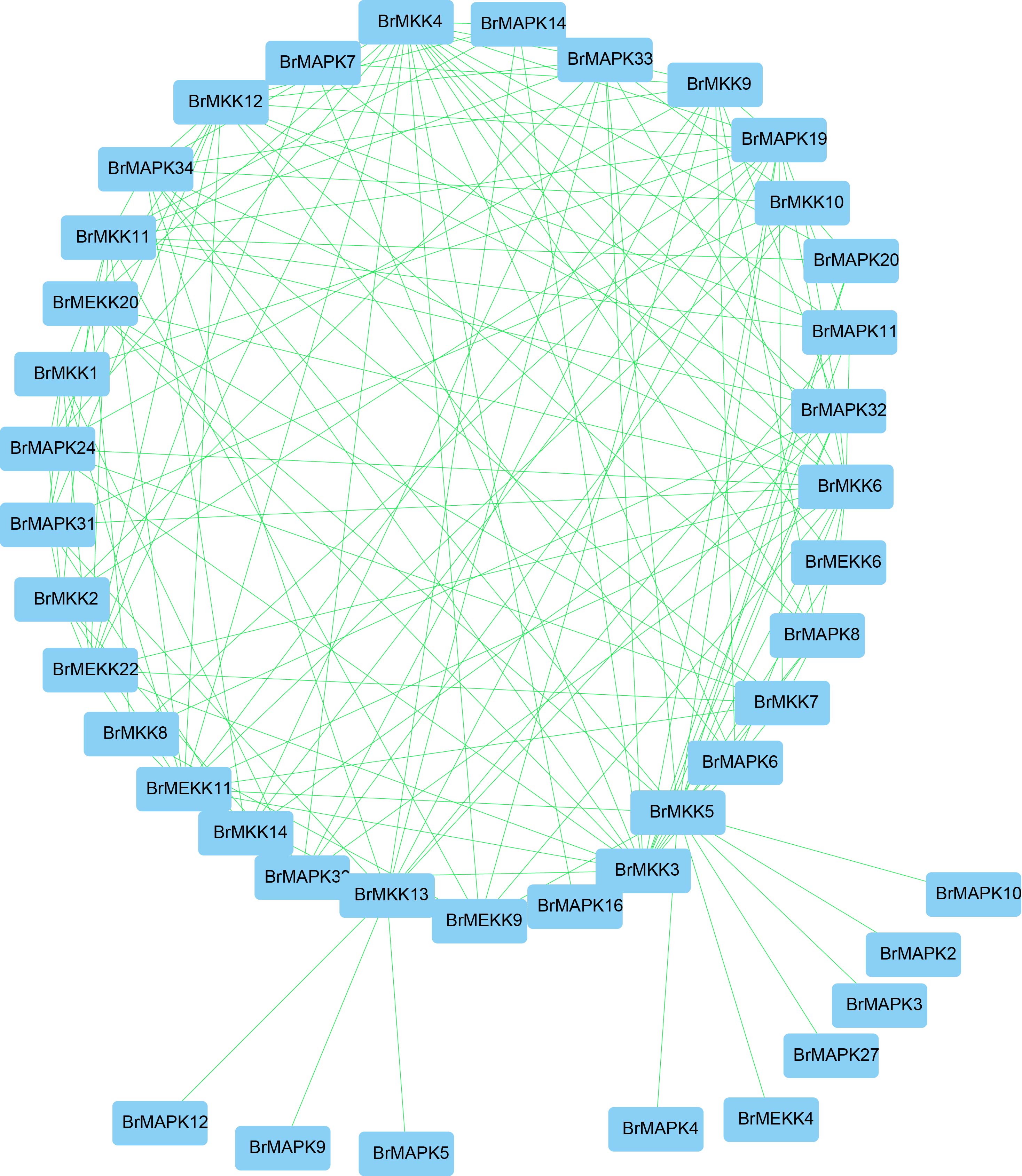

Supplement: Supplementary Figure S10 [file hortres201779-s12.jpg]

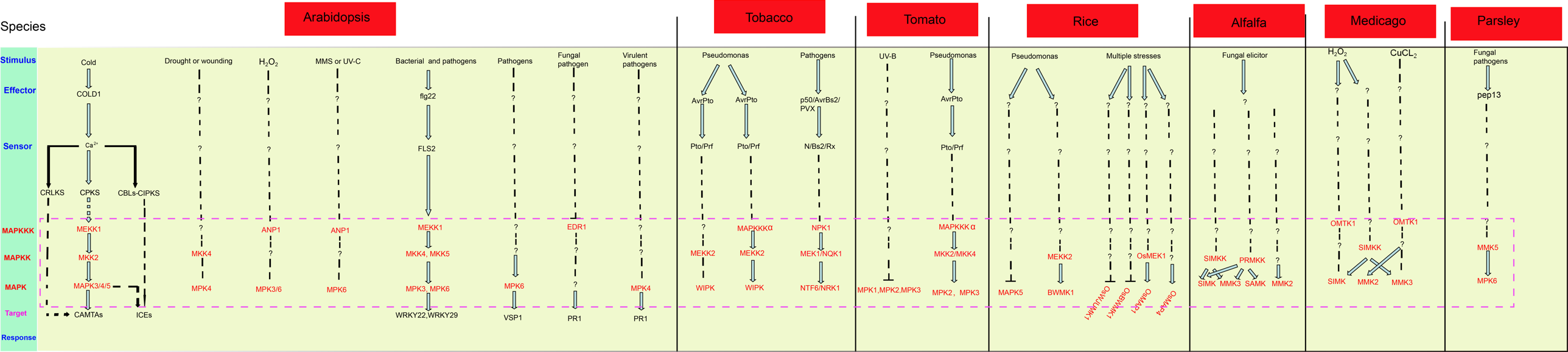

Supplement: Supplementary Figure S11 [file hortres201779-s13.jpg]

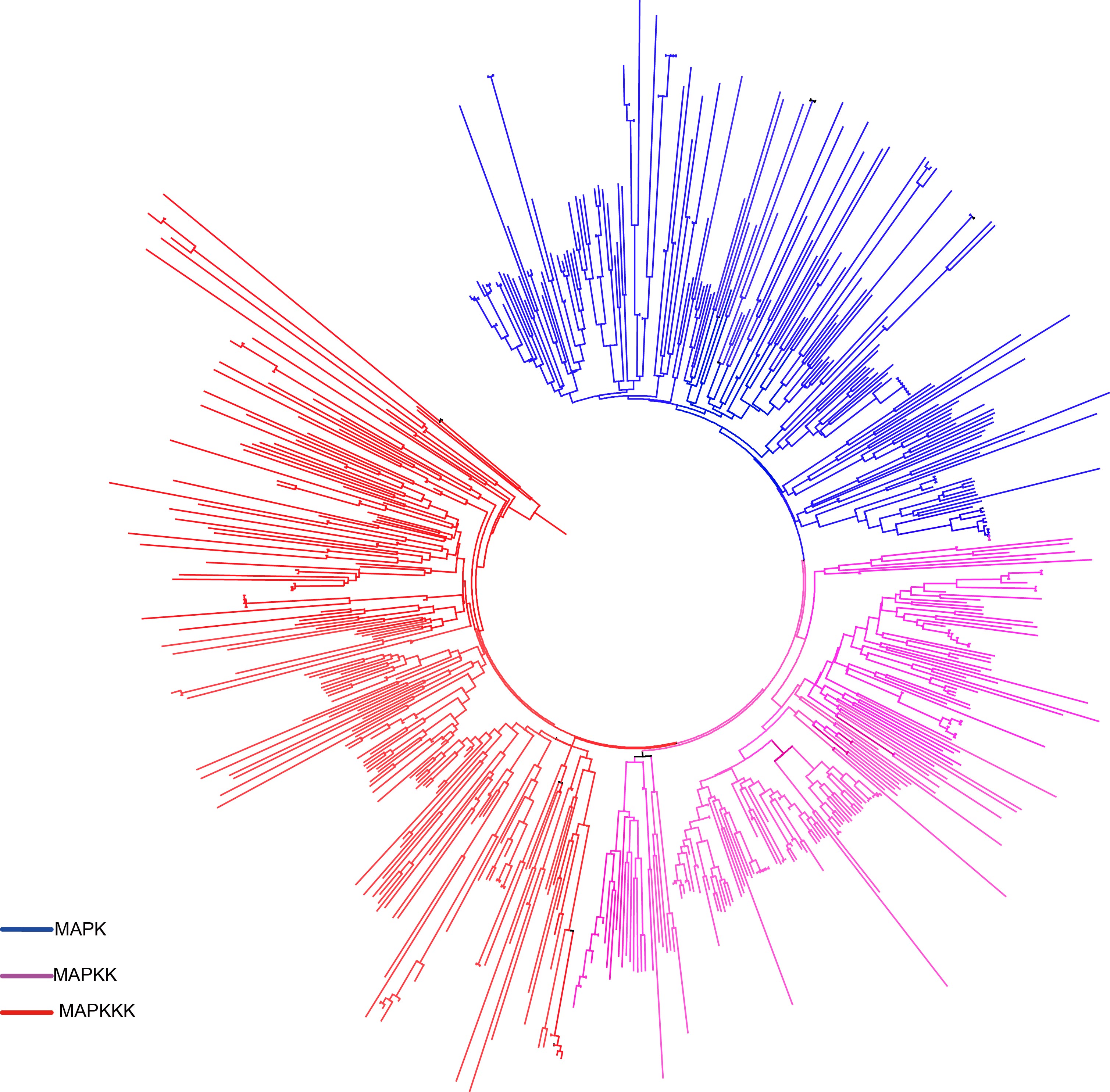

Supplement: Supplementary Figure S12 [file hortres201779-s14.jpg]
